# A Siamese Vision Transformer for Bearings Fault Diagnosis

**DOI:** 10.3390/mi13101656

**Published:** 2022-09-30

**Authors:** Qiuchen He, Shaobo Li, Qiang Bai, Ansi Zhang, Jing Yang, Mingming Shen

**Affiliations:** 1 School of Mechanical Engineering, Guizhou University, Guiyang 550025, China; 2 State Key Laboratory of Public Big Data, Guizhou University, Guiyang 550025, China; 3 School of Mechanical & Electrical Engineering, Guizhou Normal University, Guiyang 550025, China

**Keywords:** intelligent fault diagnosis, vision transformer, Siamese network, limited data, domain generation

## Abstract

Fault diagnosis methods based on deep learning have progressed greatly in recent years. However, the limited training data and complex work conditions still restrict the application of these intelligent methods. This paper proposes an intelligent bearing fault diagnosis method, i.e., Siamese Vision Transformer, suiting limited training data and complex work conditions. The Siamese Vision Transformer, combining Siamese network and Vision Transformer, is designed to efficiently extract the feature vectors of input samples in high-level space and complete the classification of the fault. In addition, a new loss function combining the Kullback-Liebler divergence both directions is proposed to improve the performance of the proposed model. Furthermore, a new training strategy termed random mask is designed to enhance input data diversity. A comparative test is conducted on the Case Western Reserve University bearing dataset and Paderborn dataset and our method achieves reasonably high accuracy with limited data and satisfactory generation capability for cross-domain tasks.

## 1. Introduction

Bearings, as core components of rotating mechanisms, are widely applied in industrial fields. Faults are highly likely to cause entire mechanical system damage and threat to the safety of employees [1,2,3,4]. Playing a crucial role in the maintenance of mechanical equipment, many fault diagnosis methods have been proposed. Traditional signal-based mechanical fault diagnosis methods commonly require manual feature extraction based on knowledge and prior experience [5]. In recent years, deep learning has made progress in many areas, such as computer vision [6,7], natural language processing [8,9] and defect detection [10,11]. Therefore, a large number of fault diagnosis methods based on deep learning have been developed. Zhao et al. [12] designed a novel intelligent fault diagnosis method for diagnosing accurately and steadily rolling bearing faults. Their approach was validated on experimental and practical bearing data. Zhang et al. [13] built a novel neural network that uses raw temporal signals as input. Their method achieved high accuracy under complex working conditions. He et al. [14] proposed a bearing fault diagnosis method based on a new strategy’s sparse auto-encoder whose weights were assigned. Hu et al. [15] proposed a new method using tensor-aligned invariant subspace learning and convolutional neural networks for cross-domain bearings fault diagnosis. Zhu et al. [16] proposed a new fault diagnosis approach based on principal component analysis and deep belief network. The time-consuming and unreliable manual feature extraction method is gradually being replaced by deep learning methods [5,17,18,19,20].

However, deep learning-based methods usually require a large amount of data for model training. Collecting a considerable amount of data for every type of failure under each working condition poses a considerable challenge in actual industrial application scenarios. Some studies of mechanical fault diagnosis have been conducted using limited data. In [21], Zhang et al. applied the Siamese network to fault diagnosis and designed a Siamese CNN model reporting good performance with limited training samples. A novel method termed meta-learning fault diagnosis framework was proposed by Li et al. [22] and performed excellently under complex working conditions. Li et al. [23] designed a deep balanced domain adaptation neural network achieving exciting results using limited labeled training data. Hang et al. [24] used principal component analysis and a two-step clustering algorithm to develop performance in a high-dimensional unbalanced training dataset. A new fault diagnosis approach based on generative adversarial network (GAN) and stacked denoising auto-encoder (SDAE) was proposed by Fu et al. [25], the experimental results representing high diagnosis accuracy under various working conditions. The Feature Space Metric-based Meta-learning Model (FSM3) was designed by Wang et al. [26] to address the challenge of limited training samples. Lu et al. [27] proposed a new cross-domain DC series fault detection framework based on Lightweight Transfer Convolutional Neural Networks. A new support vector data description based on machine learning was proposed by Duan et al. [28] for limited data. Huang et al. [29] proposed a novel method for bearings fault diagnosis under actual conditions and reported that their model achieved good performance under limited data with noise labels. Bai et al. [30] proposed a novel method for bearing fault diagnosis using multi-channel convolution neural network (MCNN) and a multiscale clipping fusion(MSCF) data augmentation algorithm to suit the challenge of limited sensor data.

At the same time, conventional learning-based methods usually assume that training data and testing data are independent and identically distributed. However, it is impractical to collect sufficient data with the same distribution of test data coming from complex work conditions. This requires the training data to cover all possible operating conditions: different working loads, speeds, noise and so on. Such strict assumptions hinder the application of intelligent fault diagnosis methods in actual industry. From a realistic perspective, the training data are usually collected from specific operating conditions, different but similar equipment, or software fault simulations, which may cause different distributions from tested data. Intelligent diagnosis techniques with a strong in-distribution assumption can fail when differences develop. In recent years, numerous research studies have produced a variety of cross-domain diagnosis methods based on transfer learning or domain adaptation employing data with inconsistencies from various source domains to break the identically distributed assumption [31,32]. These studies’ fundamental principle is to build a diagnostic model that can effectively perform in the target domain using the knowledge of the relevant source domain. Exciting performance enhancements have been made in a variety of cross-domain scenarios, such as in various work conditions [33,34] and across different equipment [19,35]. Zhang et al. [34] propose a conditional adversarial domain generalization aiming to extract domain-invariant features from the different source domains and generalize to unseen target domains. Li et al. [34] implemented adversarial domain training to extra generalized features learned from different domains to hold in new working scenarios. Zheng et al. [36] combine priori knowledge and deep domain generalization network for fault diagnosis.

Although the above methods have achieved exciting results in both research directions, studies that put limited data and domain generalization into a unified framework are rare.

In recent years, Transformer has achieved great success in natural language processing and computer vision. Ding et al. [37] applied Transformer to fault diagnosis of rolling bearings and proposed a novel method termed time–frequency Transformer which achieved satisfactory performance. Weng et al. [38] designed a one-dimensional Vision Transformer with Multiscale Convolution Fusion (MCF-1DViT) combining CNN and Vision Transformer for bearing fault diagnosis. They reported that their method can significantly improve diagnosis accuracy and anti-noise ability. Tang et al. [39] introduced integrated learning into the Vision Transformer model for bearing fault diagnosis and achieved good results. The exciting performance of these methods shows the great potential of Transformer in the field of fault diagnosis.

In the current study, we propose a novel fault diagnosis method to improve the model’s generation ability to face the two challenges, i.e., limited training data and domain generation for rolling bearings. First, the time-series signal is converted into a time-frequency graph with short-time Fourier transform (STFT). Second, a Siamese Vision Transformer (SViT) is designed to extract feature vectors efficiently and implement classification tasks. In addition, we design a new loss function, bidirectional Kullback-Liebler divergence (DKLD), to improve the performance of the proposed model. A new training strategy, i.e., the random mask, is also proposed to reduce the overfitting risk of the model. The contributions of this study include the following.

(1) The proposed SViT based on a Siamese network and ViT obtains satisfactory prediction accuracy in limited data and domain generation tasks.
(2) We obtain a new loss function by combining the KL divergence of the two directions to improve the proposed model’s performance.
(3) A novel training strategy, random mask, focusing on increasing the diversity of input data distribution is designed to enhance the generation ability of the model.
(4) The experimental result shows that the proposed method achieves effective accuracy rates and has satisfactory anti-noise and domain generation ability.

The remainder of this paper is organized as follows. Section 2 details our method, including the Siamese networks, Vision Transformer, the new loss function bidirectional KL divergence and random mask strategy. Section 3 presents the experiments, results and discussion. Finally, conclusions are drawn in Section 4.

## 2. Siamese Vision Transformer

### 2.1. The Framework of the Proposed Method

**Figure 1:**
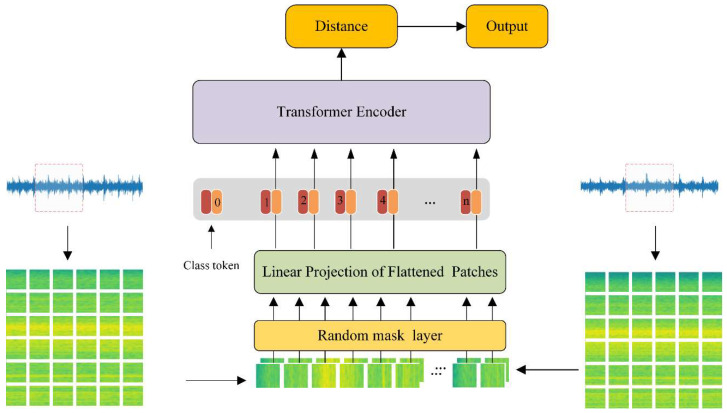
The overall framework of the proposed method.

As shown in Figure 1, the proposed method is a Siamese-based neural network using an improved vision transformer as the backbone. The inputs are a pair of time-frequency graphs obtained from raw vibration signals through STFT. First, the time-frequency graphs are divided into 8 × 8 patches. After that, the patches are fed into the Random mask layer r masking the input patches with a random rate p. Second, the 2D patches are flattened into 1D vectors through linear projection. Then the class token (a trainable vector with the same sizeas a patch) is concatenated in the font of the flattened vectors. At the same time, the positional encoders are added to the vectors. Third, the series vectors are fed into the transformer encoder constructed with two transformer encoder layers. At the top of the network, the class token outputs are used to calculate the distance of the two input time-frequency graphs. The details of the layers are shown in Table 1.

### 2.2. Data Processing

Short-time Fourier transform (STFT) uses a fixed-length nonzero window function to slide along the time axis, truncating the signal int o segments with the same length. Fourier transform can be used to obtain the local frequency spectra of the segments, assuming that these segments are stable. A 2D time-frequency graph is obtained by recombining these local frequency spectra along the time axis. The formula is presented in Equation (1).

(1) $$STFT=\int_{-\infty }^{\infty }x(t)g(t-\tau )e^{-j\omega t}dt,$$

where x(t) is the original signal and g(t−τ) is the window function applied with the center point at the time τ.

### 2.3. Siamese Network

**Figure 2:**
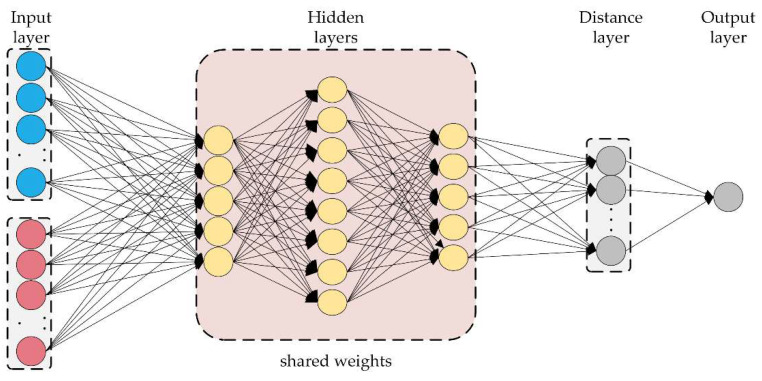
Typical Siamese network architecture.

The Siamese network algorithm was proposed by Bromley et al. [40,41] for detecting forged signatures in 1994. A typical Siamese network consists of two twin networks with the same structure and parameters. The two networks receive different inputs and are connected by an energy function calculating a metric in high-level feature space. As shown in Figure 2, tying the weights of the two subnetworks ensures that two highly similar inputs are not mapped onto extremely different positions in the feature space by their respective networks. Besides, the network is symmetrical. Thus, whenever two different inputs are presented to the twin network, the top connection layer calculates the same metric, just as the same inputs are inputted into the opposite twin network. The Siamese network can make full use of the limited training samples to achieve efficient feature extraction using the same or different sample pairs as the training samples.

As shown in Equation (2), f is the hidden layer of the model. The output layer is a fully connected layer that uses the distance feature vector as input and outputs the probability that two input data belong to the same category. This layer is obtained using Equation (3), where simg is the sigmoid function and FC represents the fully connected layer.

(2) $$d(x_{1}^{i},x_{2}^{i})=|f(x_{1}^{i})-f(x_{2}^{i})|,$$

(3) $$P(x_{1}^{i},x_{2}^{i})=simg(FC(d(x_{1}^{i},x_{2}^{i}))),$$

The network is optimized with an Adam optimizer, which adaptively sets the learning rate for each parameter.

### 2.4. Vision Transformer

**Figure 3:**
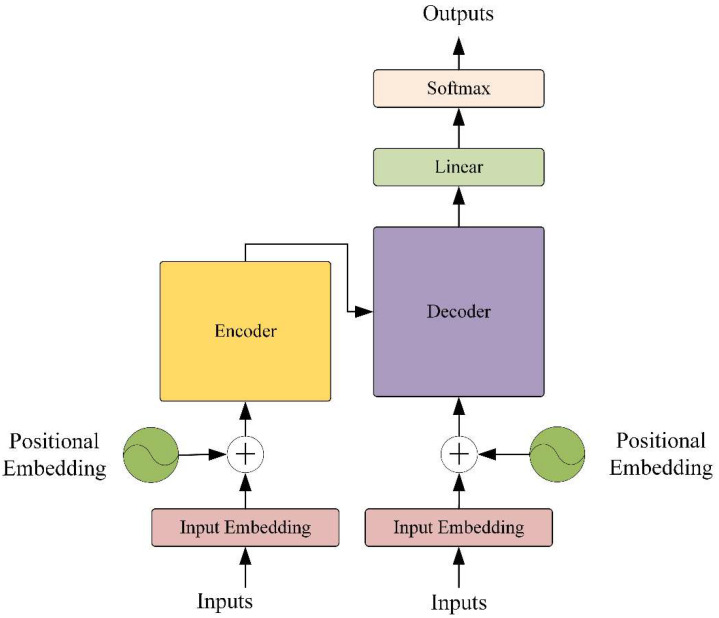
The architecture of the transformer.

A transformer is a neural network model that completely relies on a self-attention mechanism to maintain the relationship between input and output [42]. Because of the parallel architecture, which is different from the sequential structure of the traditional recurrent neural network, the transformer can consider the global information comprehensively and be trained in parallel. The architecture of the transformer model is depicted in Figure 3 and primarily comprises an encoder, a decoder and a positional embedding layer. To help the transformer address the issue of long-term dependency more effectively, positional embedding is utilized to add the relative positioning information of the input data to the data processed by the embedding layer. The transformer performs well in many time series tasks based on the above advantages. However, due to the computational complexity of the self-attention mechanism, it requires more memory and computational power in the training and prediction process. Considering the information redundancy between adjacent pixels, to reduce the computational complexity of the model the vision transformer (ViT) was proposed in [43].

Due to its global information sensing capability, ViT achieves exciting performance in the field of image and vision recognition. The structure of the ViT model consists of a projection of flattened patches, a transformer encoder and a classification head. The input image is first divided into a series of patches. These image patches are then passed through an embedding layer and output vectors of a specific length. To preserve the positional relationship of the input image, position embeddings of the same size as embedded vectors are added to the image patches. The sequence of image patches is passed to the transformer encoder, mainly composed of a multi-head attention layer and an MLP layer. The multi-head attention layer extracts different levels of self-attention information from the input through each head. The output of the class token is fed to the MLP head to give the classification result.

#### 2.4.1. Patch Embedding Layer

The Patch Embedding Layer transforms a conventional visual problem into a seq2seq problem through image segmentation and linear projection. As shown in Equation (4), suppose the input image $x\in R^{h\times w\times c}$, where h, w, c represent the image’s height, width and channel, respectively. P(*) is the dividing operation and $x_{p}\in R^{N\times (p\times p\times c)}$ denotes the sequence of the divided image, where N, p represent the number of image patches and width of a patch, respectively. L(*) is the linear projection and $x_{p}^{\prime }\in R^{N\times D}$ denotes the projected vectors, where D represent the dimension of vector space. Concat(*) is the operation of vector concatenate and $z\in R^{(N+1)*D}$ denotes the input of the transformer encoder, where cls_token is a learnable parameter with the same size as the mapped vector and the positional coders (position_coder) of the image patches are added to the vector space.

(4) $$\begin{array}{l} x_{p}=P(x)\\ x_{p}{}^{\prime }=L(x_{p})\\ z=position\_coder+(Concat(cls\_token,x_{p}^{\prime })) \end{array}$$

#### 2.4.2. Transformer Encoder

A transformer encoder layer is composed of multiple identical stacked module layers. It mainly contains two sub-layers, i.e., the multi-head self-attention layer and the MLP feedforward layer. In order to improve the stability of the model in training, each sub-layer is connected internally using residual and layer normalization.

- MLP layer

**Figure 4:**
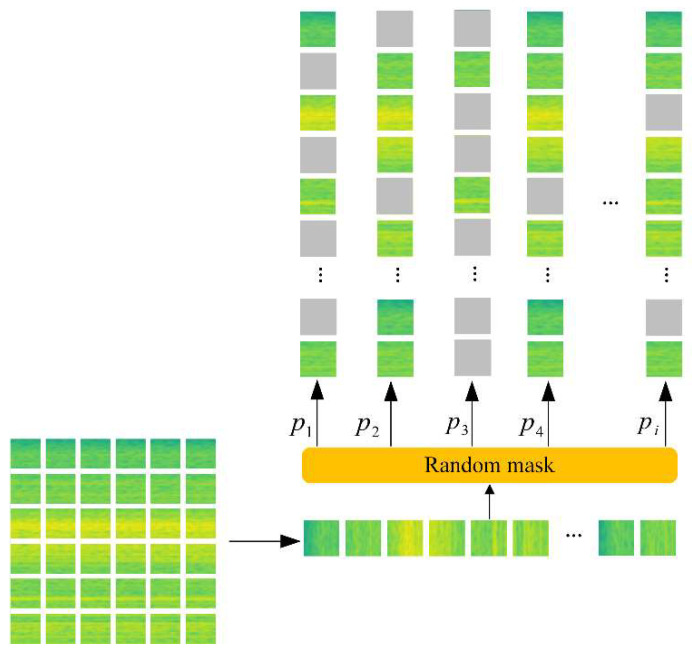
The Random mask training strategy.

The structure of the MLP is shown in Figure 4, including a fully connected layer, GELU activation function and dropout. In ViT, the Gaussian error linear unit (GELU) activation function is used in the feedforward layer. GELU activation function is expressed as Equation (5).

(5) $$GeLu(x)=x\cdot \frac{1}{2}[1+erf(\frac{x}{\sqrt{2}})]$$

- Multiheaded self-attention layer

The self-attention mechanism enables the network model to extract globally valid features, but the single-head attention mechanism can only learn the feature representation of a single representation space. In order to comprehensively extract remote features from global images, the multi-head self-attention mechanism is used to combine features from different feature subspaces.

The calculation formula of self-attention is written as Equations (6) and (7).

(6) $$Attention(Q,K,V)=soft\max(\frac{Q\cdot K^{T}}{\sqrt{d_{k}}})V,$$

(7) (Q,K,V)=XW,

where Q, K, V is the query matrix, key matrix and value matrix, respectively. These matrices are calculated by multiplying the feature matrix X with the learnable matrices W, d denotes the dimension of Q, K and V. The multi-head self-attention mechanism uses multiple self-attention heads to learn features from different representation subspaces and finally integrates these subspace features through linear mapping. The multi-head self-attention mechanism can be expressed as Equation (8).

(8) $$MultiHead(Q,K,V)=Concat(head_{1},head_{2},\cdots ,head_{n})W$$

where Concat(*) is the operation of concatenate and W denotes the weight matrix of projection.

#### 2.4.3. MLP Head

The MLP header layer consists of a fully connected layer and an activation function for the classification task of diagnosing faults. In this study, the class token vector processed by the transformer encoder is fed to the MLP header and the probability value of each fault category is obtained through the SoftMax function. The final fault category is obtained according to the maximum probability value.

### 2.5. Bidirectional KL Divergence

Kullback-Liebler (*KL*) divergence measures the similarity of a probability distribution to a reference probability distribution [44,45]. A KL divergence of 0 indicates that the two distributions are the same. For discrete probability distributions P and Q defined in the same probability space, the KL divergence [46] from Q to P is defined as Equation (9):

(9) $$DK_{KL}(P||Q)=\sum_{i}P_{i}\log\frac{P_{i}}{Q_{i}},$$

By contrast, the KL divergence from P to Q is defined as Equation (10):

(10) $$D_{KL}(Q||P)=\sum_{i}Q_{i}\log\frac{Q_{i}}{P_{i}},$$

Equations (8) and (9) clearly show that the KL divergence is asymmetric. As shown in Equation (8), in the KL divergence from *Q* to *P*, when $P_{i}=0$, regardless of the value of $Q_{i}$, $P_{i}\log\frac{P_{i}}{Q_{i}}=0$. In the two-classification problem, the loss function can only proceed to one term ($D_{KL}(P||Q)=-\log Q_{0}$ when $P_{1}=0$ or $D_{KL}(P||Q)=-\log Q_{1}$ when $P_{0}=0$). To fully measure the difference between the label and the predicted value, we design a new loss function, called bidirectional KL divergence (*DKLD*), as shown in Equation (11), where represents the label value and $Q_{i}$ is the predicting probability of the model.

(11) $$L_{DKLD}=\sum_{i}P_{i}\log\frac{P_{i}}{Q_{i}}+\sum_{i}Q_{i}\log\frac{Q_{i}}{P_{i}},$$

The iteration of gradient descent updates the parameters as shown in Equation (12):

(12) $$\begin{array}{l} W=W-\alpha \frac{\partial L_{DKLD}}{\partial W}\\ b=b-\alpha \frac{\partial L_{DKLD}}{\partial b} \end{array}$$

where W is the model’s weight, b is the bias and α is the learning rate. P is a constant and the gradient can be calculated as Equation (13).

(13) $$\frac{\partial L_{DKLD}}{\partial W}=\sum_{i}\frac{\partial Q_{i}}{\partial W}(1+\log\frac{Q_{i}}{P_{i}}-\frac{P_{i}}{Q_{i}}),$$

Compared with the gradient of the cross-entropy loss function, as shown in Equation (14), the gradient of DKLD has an additional coefficient $1+\log\frac{Q_{i}}{P_{i}}$. This coefficient contributes to the gradient regardless of whether P approaches 0 or 1. We expect that this characteristic of DKLD can help to improve the performance of the model in cases with limited training samples. To prevent calculation errors, we limit the value P to [0.001, 1] during calculations.

(14) $$\frac{\partial L_{cross\_entropy}}{\partial W}=\sum_{i}\frac{\partial Q_{i}}{\partial W}(-\frac{P_{i}}{Q_{i}}),$$

The comparison between DKLD and cross-entropy is presented in Table 2.

### 2.6. Random Mask Strategy

Similar to dropout, the mask strategy randomly deactivates neuron units in each forward propagation with probability p during training. Unlike the dropout utility neuron units, the mask strategy has larger operation granularity and the operating object in this paper is a patch. The deactivated neurons in low-level layers will affect high-level neurons. Applying mask strategy directly to the input layer can achieve the effect of data augmentation and ensemble learning at the same time. Mask is applied on input amounts to feed the input image cropped randomly and irregularly.

Masking patches with a specific distribution was not enough. Motivated by [47,48], we randomly changed the mask rate on each forward propagation to obtain a new input image with the uncertain feature. In this paper, the mask rate p∼Uniform(0.5,0.9). The visualization of the random mask strategy is illustrated in Figure 4.

## 3. Experiments, Results and Discussion

### 3.1. Experimental Setup

We set up a series of experiments to verify the prediction accuracy and generation ability of SViT on the Case Western Reserve University (CWRU) bearing datasets [49,50] and Paderborn bearing dataset [51]. The test platform is an Ubuntu 18.04, Python 3.7 and Pytorch with an Intel^®^ CORE™ i7 CPU and an Nvidia GTX 3060 GPU.

### 3.2. Comparison Models and Evaluation Metric

As shown in Table 3, the proposed model was compared with WDCNN, the Siamese CNN, PSDAN, FSM3, DeIN and HCAE. WDCNN, in which the first layer is a wide convolution kernel proposed in [24]. The Siamese CNN was designed by Zhang et al. [29]. PSDAN, FSM3, DeIN and HCAE and were proposed in [26,52,53,54], respectively. The details of the comparison methods are shown in Table 4. The SViT model was proposed by our team and the parameters of the comparison models are listed in Table 1.

Accuracy, precision, recall and F1 score are used to evaluate the performance of the proposed model. They can be obtained by the following equations:

(15) $$\mathrm{accuracy}=\frac{TP+TN}{TP+FP+FN+TN},$$

(16) $$\mathrm{precision}=\frac{TP}{TP+FP},$$

(17) $$\mathrm{recall}=\frac{TP}{TP+FN},$$

(18) $$F1=\frac{2*precision*recall}{precision+recall},$$

where TP, FP, TN, FN represent true positive, false positive, true negative and false negative, respectively.

### 3.3. Case Study 1: CWRU Bearing Datasets

**Figure 5:**
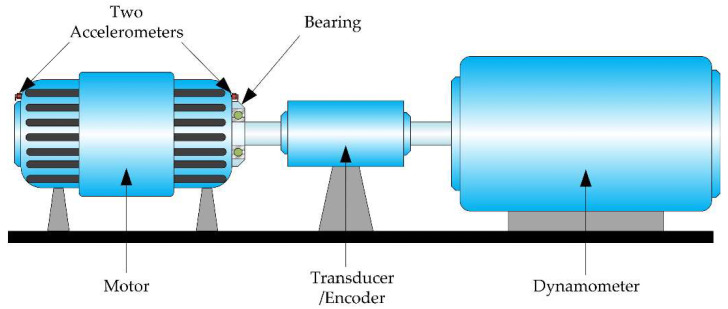
CWRU. Bearing fault diagnosis test plat.

To verify the performance of the proposed method, the 12k drive-end bearing fault data in the CWRU bearing datasets are selected as the original experimental data. Data are collected from vibration signals, as shown in Figure 5. Table 5 shows four types of faults in these data: normal, ball fault, inner race fault and outer race fault. Each fault has three subtypes: 0.007 inches, 0.014 inches and 0.021 inches. Thus, we have 10 different fault types. Each type of fault has three different loads: 1, 2 and 3 hp (with motor speeds of 1772, 1750 and 1730 RPM, respectively), as shown in Table 6. The data under different working conditions are set as domain generation experimental data. Datasets A, B and C correspond to working conditions with loads of 1, 2 and 3 hp. Each dataset contained 6000 training samples and 250 test samples, respectively.

**Figure 6:**
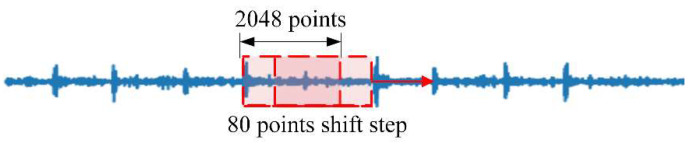
Generate sample with overlap.

We use half of the vibration signals to generate training samples and the remaining signals to generate the test set. As shown in Figure 6, the training samples are generated by a sliding window of 2048 points with 80 points of overlapping steps. The test set samples pass through sliding windows of the same size, but the samples are generated without overlapping. As shown in Table 5, the dataset includes 19,800 training samples and 750 test samples. Finally, the training and test samples of the proposed model are obtained through STFT.

#### 3.3.1. Evaluating the Effectiveness of DKLD

**Figure 7:**
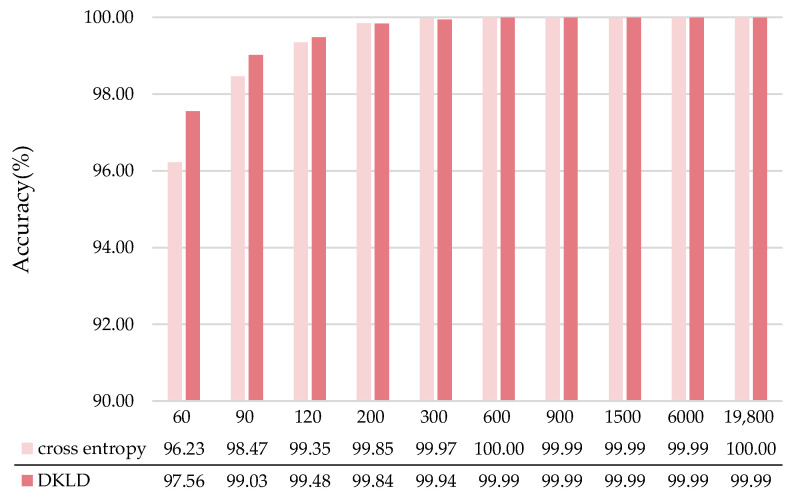
Results of proposed model training with different loss functions.

We set up a series of comparative experiments by randomly selecting 60, 90, 120, 200, 300, 600, 900, 1500, 6000 and 19,800 samples from datasets A, B and C. Each experiment uses 60% of the samples as the training set and the remaining samples as the validation set. To verify the proposed DKLD loss function’s effectiveness, we use DKLD and cross-entropy to train our model separately with different samples size and then compare the test results. As shown in Figure 7, in the cases with a small number of training samples, DKLD significantly improves the model’s performance compared with that of cross-entropy. For example, when the sample size is 60 and 90, the accuracy rates of using DKLD are 1.33% and 0.56% higher than that of using cross-entropy, respectively. When the training sample size is increased to 120 and above, the performance of the two-loss functions is exceptionally close, reaching more than 99%.

**Figure 8:**
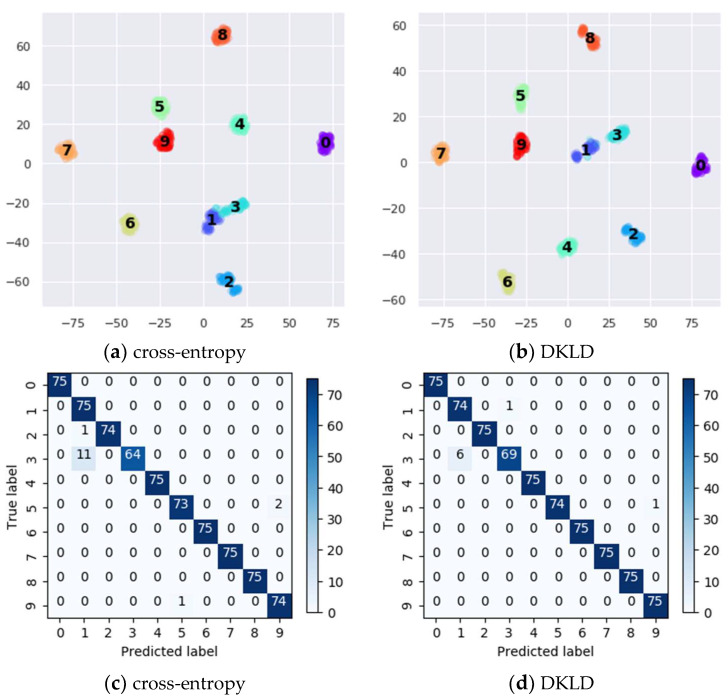
Feature visualization via t-SNE (**a**,**b**) and confusion matrix (**c**,**d**).

To improve the understanding of the effect of DKLD, we use t-distributed stochastic neighbor embedding (t-SNE) to visualize the output of the last hidden fully connected layer of the model trained with DKLD and cross-entropy in 60 sample sizes. As shown in Figure 8a,b, the features of DKLD are more divisible than cross-entropy, particularly in the 1 and 3 categories. Figure 8c,d shows the confusion matrix of the results.

#### 3.3.2. The Effect of the Number of Transformer Encoder Layers

**Figure 9:**
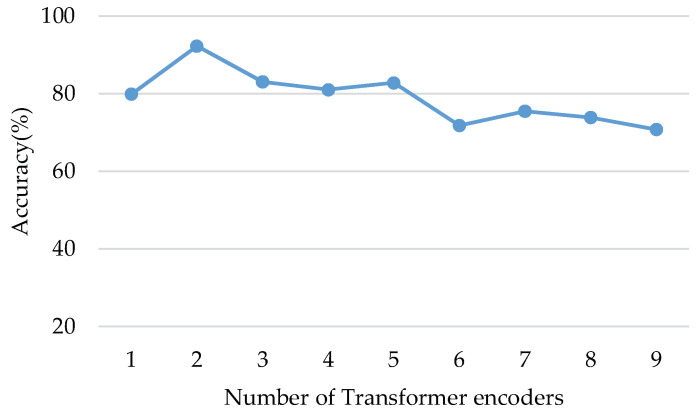
The accuracy of the proposed model with different numbers of transformer encoders.

To observe the effect of the number of transformer encoders, we tested the performance of the proposed model with the different number of transformer encoders in the cross-domain experiment from dataset C to dataset A (the most difficult cross-domain task [21]). As shown in Figure 9, the proposed model achieved the best performance with two transformer encoders. SViT with two transformer encoders is implemented in follow-up experiments.

#### 3.3.3. Ablation Experiments

To verify the effectiveness of the Random Mask strategy and Siamese network structure, we set up ablation experiments on cross-domain with 600 training samples. The proposed method is removed the Random mask strategy and Siamese network structure in turn. When the Siamese network structure is removed, the distance layer is instead of a fully connected classifier.

As shown in Table 7, (*w/o*) means without. It can be seen that the Random mask strategy and the Siamese network effectively improve the robustness of the model in cross-domain tasks.

#### 3.3.4. Comparison of Results with Different Samples Sizes

Implementing the same experimental setup as above, we evaluate the performance of various methods by using different numbers of training samples. We repeat the sample selection process five times for each sample size to generate different training sets to reduce the bias when randomly selecting a small training set. For each random training sample set, we repeat the algorithm training four times to address the randomness of the algorithm. Each series of experiments is repeated 20 times. We use one-shot testing in the Siamese CNN and our method.

**Figure 10:**
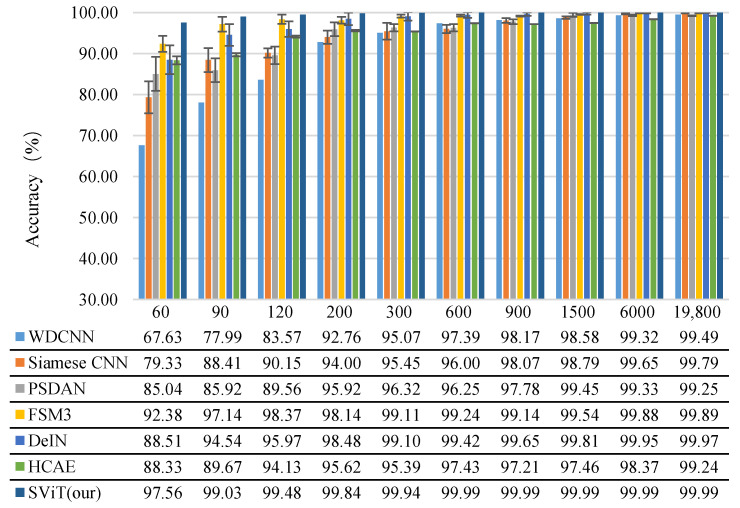
Diagnosis results of the proposed method compared with those of the comparison models.

Figure 10 clearly shows that as the amount of training samples increases, the accuracy of all methods also increases, but their standard deviation decreases. This shows the sensitivity of the intelligent fault diagnosis method based on deep learning to the amount of training data.

Subsequently, we check whether the proposed SViT model’s accuracy is better than those of the other models in the cases with limited training samples (e.g., 60 and 90). In both cases, our model performs better than the other models. Simultaneously, the experimental results indicate that when the training sample size is increased to 900 and above, all the algorithms’ performance becomes increasingly similar and their accuracy rates are all higher than 97%. This comparison proves that the proposed SViT exhibits significant advantages over the comparison algorithms in cases with limited training samples. Even in the case of 60 training samples, the proposed algorithm’s accuracy rate still reaches 97.56%.

#### 3.3.5. Performance in Noisy Environment

In this experiment, we evaluate the performance of the proposed model in a noisy environment. The model is trained with raw data and then tested with samples added with white Gaussian noise with different signal-to-noise ratios (*SNR*s). SNR is defined as the ratio of the signal power to the noise power and it is frequently expressed in decibels (dB), as follows:$SNR_{dB}=10\log_{10}(\frac{P_{signal}}{P_{niose}})$, where $P_{signal}$ denotes the power of the signal and $P_{niose}$ indicates the power of noise. The SNR range is from −4dB to 10dB. The higher the SNR value, the stronger the intensity of noise.

**Figure 11:**
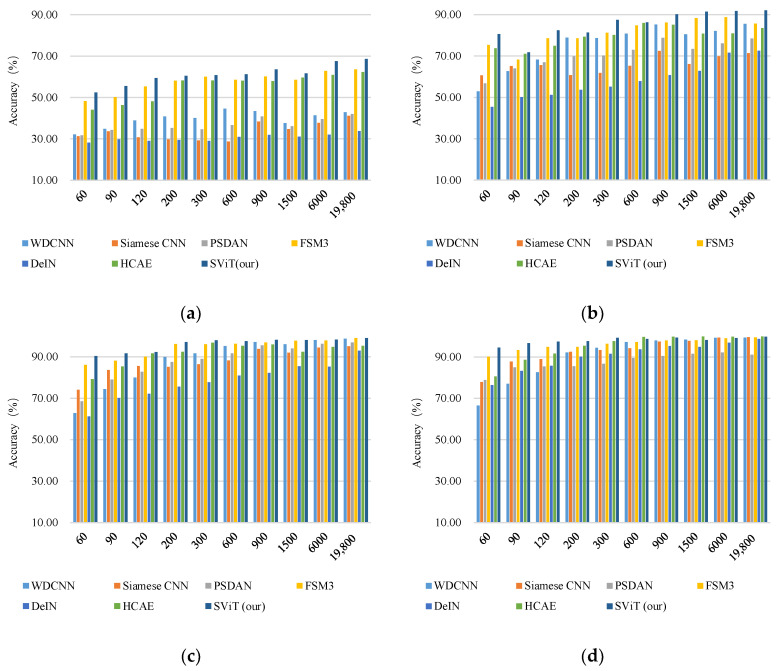
Results of different sample sizes in a noisy environment. (**a**) SNR = −4; (**b**) SNR = 0; (**c**) SNR = 4; (**d**) SNR = 8.

In Figure 11, we examine the effect of training sample size on the test accuracy of each model in different noisy environments. In Figure 11a,b, SNR = −4 and 0 represent substantial noise interference. By contrast, in Figure 11c,d, SNR = 4 and 8 represent weak noise interference. The anti-noise capability of the proposed model is better than those of the other models. In particular, the advantage is more apparent in cases with intense noise, as shown in Figure 11a,b. Considering that the proposed method is not specifically designed to improve the anti-noise, according to the report in [21], we speculate that this anti-noise ability is derived from the twin network structure.

#### 3.3.6. Domain Generation Experiments

To further verify the domain generalization ability of the proposed model, we conduct a cross-domain experiment where all models are trained in the source domain and tested in the target domain. It should be noted that the model does not touch the target domain data during training. The experiment was repeated five times for each task. The results of the cross-domain tasks were observed. The classification accuracies of the experiment are shown in Table 8, in which A-B refers to training on dataset A and testing on dataset B. The proposed SViT achieved the best performance among all the methods in all the scenarios. Specifically, SViT achieved an accuracy of 92.24% in C-A task (the most difficult task), which was 13.4%, 31.88%, 12.86%, 2.8%, 12.56% and 11.57% higher than WDCNN, Siamese CNN, PSADAN, FSM3, DeIN and HCAE, respectively. This shows that the proposed method performs better domain generalization than the comparison methods. Table 9, Table 10 and Table 11 demonstrate precision, recall and F1 score compressions for cross domain task C-A with 6000 training samples. The results show that the proposed SViT outperformed all of the compared approaches.

**Figure 12:**
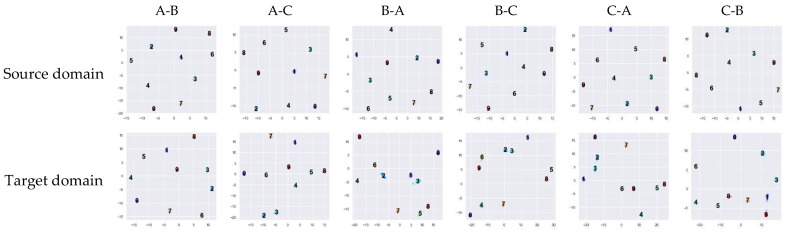
Feature visualization via t-SNE in cross-domain tasks.

To further understand cross-domain generation ability, the encoded feather of source domain data and target domain data in different cross-domain task are investigated. T-distributed stochastic neighbor embedding (t-SNE) is used to visualize the output of the class token of the model training with 6600 training samples in the source domain, as shown in Figure 12.

### 3.4. Case Study 2: Paderborn Dataset

#### 3.4.1. Data Description

**Figure 13:**
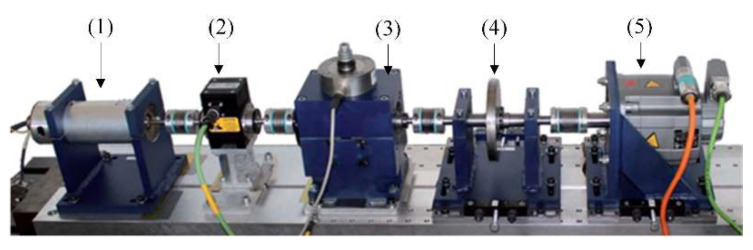
Test rig of Paderborn bearing dataset.

As shown in Figure 13, there are five modules the Paderborn dataset test rig [51]: (1) electric motor, (2) torque-measurement shaft, (3) rolling bearing test module, (4) flywheel and (5) load motor. Bearings are installed in the test module to collect experimental data. Fault types of bearings include artificial and real damage.

There work conditions are selected to obtain different domain datasets. In dataset D, the test platform runs at *n* = 1500 rpm with a load torque of *M* = 0.7 Nm and a radial force on the bearing of *F* = 1000 N. In dataset E, load torque changes to *M* = 0.1. In dataset F, radial force changes to *F* = 400 N. The details of three datasets are shown in Table 12.

In the experiment, datasets contain vibration signals obtained from healthy, artificially damaged bearings and naturally damaged bearings. The datasets filenames selected are shown in Table 13. The details of the datasets selected are in Table 14.

#### 3.4.2. Results and Analysis

**Figure 14:**
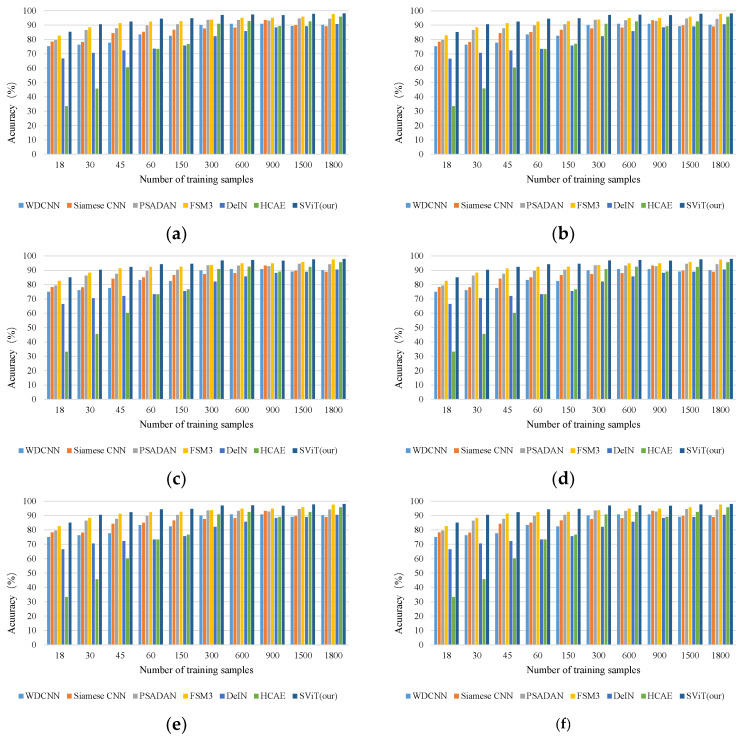
The mean accuracy of cross-domain task with the different number of training samples on the Paderborn dataset. (**a**) D-E; (**b**) D-F; (**c**) E-D; (**d**) E-F; (**e**) F-D; (**f**) F-E.

Performing the same implementation, Figure 14 shows the cross-domain tasks accuracy of comparison approaches and our method with the increasing number of training samples. The results show that our method outperformed the state-of-the-art methods in all the scenarios.

Table 15 reports the cross-domain tasks accuracy of different methods with 1800 training samples. The proposed method outperformed all comparative methods by 1.80–4.29% on average. Table 16, Table 17 and Table 18 compare the methods in precision, recall and F1 score in the cross-domain task E-D with 1800 training samples. The results also show that our method superior to the alternatives.

## 4. Conclusions

In this work, an intelligent bearing fault diagnosis method, i.e., SViT has been proposed to face the challenges coming from limited data and domain generation. We have designed a Siamese Vision transformer (SViT) to extract features efficiently. In addition, a loss function called DKLD has been proposed to improve our model’s prediction accuracy and generation capability. Furthermore, a novel random mask training strategy has been conducted with the SViT to reduce the overfitting risk and improve the model’s generation ability. We present the experimental results showing that our method has better generalization ability in the limited data and cross-domain tasks compared with the state-of-the-art approaches.

However, the proposed method in this paper still has some restrictions. For instance, this method is limited to cross-domain tasks on the same equipment. In addition, In the prediction stage of SViT, a little more supporting data in the target domain is still required, which limits the application scenarios of the proposed method.

## Figures and Tables

**Table 1:** Details of the proposed model.

NO.	Layer Type	Input Size	Output Size
		Size/Stride	(Width × Depth)
1	Input	/	64 × 64 × 1
2	Patch layer	64 × 64 × 1	8 × 8 × 64
3	Patch Flatten	8 × 8 × 64	64 × 64
4	Fully-connected	64 × 64	32 × 64
5	Class torken &position endoer	32 × 64	32 × 65
6	Transformer Encoder	32 × 65	32 × 65
7	Transformer Encoder	32 × 65	32 × 65
8	Fully-connected	32 × 1	1

**Table 2:** Comparison between DKLD and cross-entropy.

	Cross-Entropy	DKLD
Equation	$\sum_{i}P_{i}\log(\frac{1}{Q_{i}})$	$\sum_{i}P_{i}\log(\frac{P_{i}}{Q_{i}})+\sum_{i}Q_{i}\log(\frac{Q_{i}}{P_{i}})$
Gradient	$\sum_{i}\frac{\partial Q_{i}}{\partial W}(-\frac{P_{i}}{Q_{i}})$	$\sum_{i}\frac{\partial Q_{i}}{\partial W}(1+\log\frac{Q_{i}}{P_{i}}-\frac{P_{i}}{Q_{i}})$

**Table 3:** The comparison methods.

Input Type	Method Name	Implementation Details
Time-based	WDCNN	Details referred to [55].
	Siamese CNN	Details referred to [21].
	PSDAN	Implementation details referred to [52].
	FSM3	Details referred to [26].
Time-Frequency	DeIN	Details referred to [53].
	HCAE	Implementation details referred to [54]
	SViT (our)	As shown in Table 1.

**Table 4:** Details of the comparison methods.

Layers	WDCNN (Kernel Size/Stride)	Siamese CNN (Kernel Size/Stride)	PSDAN (Kernel Size/Stride)	FSM3 (Kernel Size/Stride)	DeIN (Kernel Size/Stride)	HCAE (Kernel Size/Stride)
1	Convolution (64 × 16/16)	Convolution (64 × 16/16)	Convolution (128 × 32/1)	Convolution (64 × 1/16)	Convolution (2 × 2 × 64/2)	Convolution (3 × 3 × 16/2)
2	Pooling (2 × 16/2)	Pooling (2 × 16/2)	Pooling (4 × 32/4)	Pooling (2 × 1/2)	Offset_low (3 × 3)	Convolution (3 × 3 × 32/2)
3	Convolution (3 × 32/1)	Convolution (3 × 32/1)	Convolution (32 × 64/1)	Convolution (3 × 1/1)	Inception_Resnet 16	Convolution (3 × 3 × 32/2)
4	Pooling (2 × 32/1)	Pooling (2 × 32/1)	Pooling (4 × 64/4)	Pooling (2 × 1/2)	Reduction	Convolution (3 × 3 × 32/2)
5	Convolution (3 × 64/1)	Convolution (3 × 64/1)	Convolution (8 × 128/1)	Convolution (3 × 1/1)	Offset_pooling (3 × 3)	Flatten layer
6	Pooling (2 × 64/2)	Pooling (2 × 64/2)	Pooling (4 × 128/4)	Pooling (2 × 1/2)	Pooling (3 × 3/1)	Fully-connected (512 × 64)
7	Convolution (3 × 64/1)	Convolution (3 × 64/1)	Convolution (3 × 128/1)	Convolution (3 × 1/1)	Convolution (1 × 1/1)	Fully-connected (64 × 32)
8	Pooling (2×64/2)	Pooling (2×64/2)	Pooling (4 × 128/4)	Pooling (2 × 1/2)	Dropout	Classifier (fully-connectied-Softmax) (32 × 10)
9	Convolution (3 × 64/1)	Convolution (3 × 64/1)	Convolution (3 × 128/1)	Convolution (3 × 1/1)	Offset_top (3 × 3)	Transposed convolution (3 × 3 × 32/2)
10	Pooling (2 × 64/2)	Pooling (2 × 64/2)	Pooling (4 × 128/4)	Flatten	GlobalMax_Pooling	Transposed convolution (3 × 3 × 32/2)
11	Flatten-layer	Flatten-layer	Flatten-layer	Fully Connected	Softmax	Transposed convolution (3 × 3 × 32/2)
12	Fully-connected (192 × 100)	Fully-connected (192 × 100)	Fully-Connected (512 × 256)	Convolution (3 × 1/1)	Inception-resnet8	Transposed convolution (3 × 3 × 16/2)
13	Fully-connected (100 × 10)	Distance layer	Fully-Connected (256 × 128)	Convolution (3 × 1/1)	Reduction	Reconstruction
14	-	Fully-connected (100 × 1)	Fully-Connected (128 × 10) (128 × 2)	Flatten	Inception-resnet4	-
15	-	-	_	Fully Connected	Dropout	-
16	-	-	_	-	Convolution (2 × 2/1)	-
17	-	-	_		Offset_top	-
18	-	-	_		Pooling	-
19	-	-	_		softmax	-

**Table 5:** Description of CWRU dataset.

Fault Location		None	Ball			Inner Race			Outer Race			Load
Fault Diameter (inch)		0	0.007	0.014	0.021	0.007	0.014	0.021	0.007	0.014	0.021	
Class Labels		1	2	3	4	5	6	7	8	9	10	
Dataset A	Train	600	600	600	600	600	600	600	600	600	600	1
	Test	25	25	25	25	25	25	25	25	25	25	
Dataset B	Train	600	600	600	600	600	600	600	600	600	600	2
	Test	25	25	25	25	25	25	25	25	25	25	
Dataset C	Train	600	600	600	600	600	600	600	600	600	60 0	3
	Test	25	25	25	25	25	25	25	25	25	25	

**Table 6:** Three different working conditions.

Datasets	Load/HP	Rotational Speed/rpm	Damage Size/10^−3^ in.
A	1	1772	7, 14, 21
B	2	1750	7, 14, 21
C	3	1730	7, 14, 21

**Table 7:** Ablation experiments with 600 training samples.

Methods	A-B	A-C	B-A	B-C	C-A	C-B	Average
SViT	97.35	93.64	95.42	97.76	88.75	93.31	94.37
(*w/o*) Random mask	94.89	87.26	85.67	90.14	87.64	82.75	88.06
(*w/o*) Siamese network	95.13	91.73	92.11	95.82	86.46	92.15	92.23
(*w/o*) Random mask &Siamese network	92.01	82.41	81.43	87.21	78.82	80.21	83.68

**Table 8:** Mean classification accuracy (%) with 6000 training samples on CWRU.

Methods	A-B	A-C	B-A	B-C	C-A	C-B	Average
WDCNN	97.08	91.48	93.00	91.80	78.84	85.88	89.68
Siamese CNN	99.24	90.40	88.28	90.12	60.36	65.36	82.29
PSADAN	98.10	92.67	90.67	90.86	79.38	92.37	90.68
FSM3	98.14	91.54	93.54	97.36	89.44	96.24	94.38
DeIN	93.14	70.76	76.33	83.17	79.68	76.56	79.94
HCAE	98.67	82.67	89.37	90.37	80.67	76.34	86.35
SViT (our)	99.54	93.82	94.24	99.85	92.24	98.78	96.41

**Table 9:** Precision (%) comparison for cross-domain task C-A with 6000 training samples on CWRU.

	Class 1	Class 2	Class 3	Class 4	Class 5	Class 6	Class 7	Class 8	Class 9	Class 10
WDCNN	76.67	78.60	79.17	79.47	77.67	79.41	79.93	76.49	81.27	80.13
Siamese CNN	58.30	58.53	58.79	61.97	58.78	64.67	63.10	59.08	59.94	60.54
PSADAN	75.96	83.21	80.20	79.00	79.47	81.46	77.05	80.07	78.71	79.34
FSM3	88.16	91.90	92.39	92.18	86.82	87.42	89.84	88.45	89.93	88.06
DeIN	79.04	81.63	80.20	77.53	81.51	77.78	81.31	80.00	79.80	78.55
HCAE	78.21	82.56	79.19	80.67	82.23	78.48	85.32	82.90	78.07	79.80
SViT (our)	91.30	92.47	93.40	93.16	93.46	91.45	93.29	90.20	93.96	90.07

**Table 10:** Recall (%) comparison for cross-domain task C-A with 6000 training samples on CWRU.

	Class 1	Class 2	Class 3	Class 4	Class 5	Class 6	Class 7	Class 8	Class 9	Class 10
WDCNN	76.67	78.33	82.33	80.00	77.67	81.00	79.67	77.00	76.67	79.33
Siamese CNN	55.00	58.33	61.33	63.00	58.00	64.67	61.00	59.67	62.33	60.33
PSADAN	79.00	77.67	78.33	79.00	80.00	82.00	78.33	77.67	81.33	80.67
FSM3	89.33	87.00	89.00	90.33	90.00	88.00	91.33	89.33	89.33	91.00
DeIN	76.67	80.00	78.33	81.67	79.33	79.33	78.33	80.00	80.33	83.00
HCAE	81.33	77.33	78.67	80.67	78.67	82.67	83.33	85.67	78.33	80.33
SViT (our)	91.00	90.00	89.67	95.33	95.33	92.67	92.67	92.00	93.33	90.67

**Table 11:** F1 score (%) comparison for cross-domain task C-A with 6000 training samples on CWRU.

	Class 1	Class 2	Class 3	Class 4	Class 5	Class 6	Class 7	Class 8	Class 9	Class 10
WDCNN	76.67	78.46	80.72	79.73	77.67	80.20	79.80	76.74	78.90	79.73
Siamese CNN	56.60	58.43	60.03	62.48	58.39	64.67	62.03	59.37	61.11	60.43
PSADAN	77.45	80.34	79.26	79.00	79.73	81.73	77.69	78.85	80.00	80.00
FSM3	88.74	89.38	90.66	91.25	88.38	87.71	90.58	88.89	89.63	89.51
DeIN	77.83	80.81	79.26	79.55	80.41	78.55	79.80	80.00	80.07	80.71
HCAE	79.74	79.86	78.93	80.67	80.41	80.52	84.32	84.26	78.20	80.07
SViT (our)	91.15	91.22	91.50	94.23	94.39	92.05	92.98	91.09	93.65	90.37

**Table 12:** Working conditions of test bearing on Paderborn dataset.

Datasets	Rotational [rpm]	Load Torque [Nm]	Radial Force [N]	Name of Setting
D	1500	0.7	1000	N15_M07_F10
E	1500	0.1	1000	N15_M01_F10
F	1500	0.7	400	N15_M07 _F04

**Table 13:** Data sets used for experiments.

Fault Location	None	Out Race	Inner Race
File NO.	K001 K002	Artificial (KA01)	Artificial (KI01)
		Real damages (KA04)	Real damages (KI14)

**Table 14:** Detail of datasets on Paderborn.

Dates Sets	Splitting	None (Class 1)	Inner Race (Class 2)	Out Race (Class 3)
D	Training	600	600	600
	Testing	40	40	40
E	Training	600	600	600
	Testing	40	40	40
F	Training	600	600	600
	Testing	40	40	40

**Table 15:** Mean classification accuracy (%) with 1800 samples on the Paderborn dataset.

Methods	D-E	D-F	E-D	E-F	F-D	F-E	Average
WDCNN	90.13	97.5	94.99	93.33	95.83	91.16	93.82
Siamese CNN	88.98	95.83	95.83	92.5	96.13	88.19	92.91
PSADAN	94.26	92.82	97.42	95.33	96.01	90.24	94.35
FSM3	97.57	98.04	99.45	99.14	96.89	94.68	97.62
DeIN	90.53	98.12	91.77	89.82	98.24	94.55	93.84
HCAE	95.67	96.84	99.67	96.26	95.76	93.67	96.31
SViT (our)	98.03	98.06	99.83	99.33	97.06	96.34	98.11

**Table 16:** Precision (%) comparison for cross-domain task E-D with 1800 training samples per class on the Paderborn dataset.

	Class 1	Class 2	Class 3
WDCNN	78.64	79.73	78.31
Siamese CNN	59.21	61.02	61.03
PSADAN	81.51	76.66	80.10
FSM3	89.68	89.86	88.80
DeIN	80.30	79.03	79.83
HCAE	80.64	81.15	80.39
SViT (our)	92.84	92.36	91.65

**Table 17:** Recall (%) comparison for cross-domain task E-D with 1800 training samples per class on the Paderborn dataset.

	Class 1	Class 2	Class 3
WDCNN	79.17	78.67	78.83
Siamese CNN	62.17	57.67	61.33
PSADAN	80.83	78.83	78.50
FSM3	89.83	88.67	89.83
DeIN	80.17	79.17	79.83
HCAE	79.83	79.67	82.67
SViT (our)	90.83	92.67	93.33

**Table 18:** F1 score (%) comparison for cross-domain task E-D with 1800 training samples per class on the Paderborn dataset.

	Class 1	Class 2	Class 3
WDCNN	78.90	79.19	78.57
Siamese CNN	60.65	59.30	61.18
PSADAN	81.17	77.73	79.29
FSM3	89.76	89.26	89.31
DeIN	80.23	79.10	79.83
HCAE	80.23	80.40	81.51
SViT (our)	91.83	92.51	92.49

## Data Availability

The data used to support this study are available at the websites https://engineering.case.edu/bearingdatacenter/download-data-file and https://mb.uni-paderborn.de/kat/forschung/datacenter/bearing-datacenter, accessed on 10 August 2022.
